# *Gastrodia elata* Blume Rhizome Aqueous Extract Improves Arterial Thrombosis, Dyslipidemia, and Insulin Response in Testosterone-Deficient Rats

**DOI:** 10.1155/2017/2848570

**Published:** 2017-05-21

**Authors:** Min Jung Kim, Hye Jeong Yang, Bo Reum Moon, Ji Eun Kim, Kang Sung Kim, Sunmin Park

**Affiliations:** ^1^Division of Nutrition and Metabolism Research, Korea Food Research Institute, Gyeonggi-do, Republic of Korea; ^2^Department of Food Science and Nutrition, Yong In University, Gyeonggi-do, Republic of Korea; ^3^Division of Strategic Food Industry Research, Korea Food Research Institute, Gyeonggi-do, Republic of Korea; ^4^Department of Food and Nutrition, Obesity/Diabetes Center, Hoseo University, Asan, Republic of Korea

## Abstract

Testosterone deficiency deteriorates glucose and lipid metabolism with reducing muscle mass. We investigated whether the consumption of water extracts of* Gastrodia elata *Blume rhizome (GEB) rich in gastrodin would reduce the symptoms of testosterone deficiency and improve blood flow in orchidectomized (ORX) rats. ORX rats were given high-fat diets supplemented with either 1% cellulose (ORX-control), 0.3% GEB (GEB-L), or 1% GEB (GEB-H) for 8 weeks. Sham-operated rats were fed the same diet as OVX-control rats (normal-control). ORX-control rats had reduced serum testosterone levels by one-fifth, compared to normal-control rats. ORX-control rats exhibited decreased lean body mass, attenuated blood flow, and impaired cholesterol metabolism and glucose control due to decreased insulin secretory response. GEB increased serum insulin levels dose-dependently and GEB-H mostly enhanced dyslipidemia in ORX rats. GEB completely normalized arterial thrombosis time and blood flow in ORX rats. Interestingly, ORX-control rats showed attenuated hepatic insulin signaling but greater AMPK and CREB activities, which reduced triglyceride accumulation, compared to normal-control. GEB-H improved hepatic insulin signaling but maintained the AMPK and CREB activities in ORX rats. In conclusions, GEB ameliorated the impairment of cholesterol and glucose metabolism and blood flow in ORX rats. GEB may be a potential preventive measure for reducing the risk of cardiovascular diseases associated with testosterone deficiency.

## 1. Introduction

Aging is accompanied by a gradual and modest decline in the levels of blood testosterone in men. Indeed, these levels can drop as much as two-thirds over a lifetime. The symptoms of testosterone deficiency are decreased libido, vasomotor instability, bone mineral density, and muscle strength and mass, as well as depression and erectile dysfunction. It also reduces blood flow, induces vasodilation, and exacerbates the progression of chronic diseases [1]. In epidemiological studies, it has been shown that lower testosterone is positively associated with a higher incidence of cardiovascular diseases (e.g., atherosclerosis and coronary artery diseases) [2]. It may be associated with reducing muscle mass to develop sarcopenia. Sarcopenia increases the risk of cardiovascular diseases especially stroke in adult population [3]. Testosterone treatment increases coronary blood flow, modestly increases muscle mass, and reduces fat mass. However, there is not sufficient evidence that testosterone treatment reduces the incidence of cardiovascular diseases in those with low testosterone [4]. The efficacy of testosterone replacement treatment in patients with cardiovascular diseases remains controversial. In June 2015, the FDA announced that “testosterone treatment is indicated for replacement therapy only in males with conditions associated with a deficiency in endogenous testosterone, specifically primary hypogonadism or hypogonadotropic hypogonadism” [5]. Therefore, alternative therapy may be required for treating the symptoms of testosterone deficiency.

*Gastrodia elata* Blume (GEB; Orchidaceae, Tian ma) is mainly distributed in the mountainous areas of eastern Asia, such as China, Korea, Japan, and India. Its rhizome is extensively used in traditional Chinese herbal medicine to treat brain-related diseases (e.g., headache, migraine, dizziness, epilepsy, infantile convulsion, Alzheimer's disease, and stroke). The rhizome of GEB is an approved food additive according to the FDA's “Everything Added to Food in the United States” database [6]. Li et al. [7] identified a total of 64 compounds in the rhizome. The major components with neuropharmacological properties are gastrodin, 4-hydroxybenzaldehyde, 4-HBA, vanillin, and vanillyl alcohol [8]. The rhizome also has some known antiobesity properties. Specifically, it reduces insulin resistance in diet-induced obese rats by decreasing fat accumulation in adipocytes. It does this by activating fat oxidation and potentiating leptin signaling (mainly through the actions of 4-hydroxybenzaldehyde and vanillin) [9]. The brain, particularly the hypothalamus, is important for regulating energy and glucose metabolism [10, 11]. Likewise, testosterone secretion is regulated by the hypothalamus-pituitary-testes axis. As such, GEB may have a positive impact on the symptoms of testosterone deficiency. However, no studies have investigated the effects of GEB on blood flow or the factors that affect blood flow (e.g., energy, glucose, lipid metabolism, and body composition).

In the present study, we hypothesized that the long-term consumption of water extracts of the rhizomes GEB would reduce the symptoms of testosterone deficiency and improve blood flow in testosterone-deficient animals with diet-induced obesity. We also examined the associated mechanisms of actions. We performed orchidectomy on male rats and fed them a high-fat diet supplemented with water extracts of GEB rhizome.

## 2. Materials and Methods

### 2.1. Total Phenol and Flavonoid Contents in Water Extract of GEB Rhizomes

Rhizomes of GEB were provided by the Muju* Gastrodia elata* Blume Cooperative Production Co. (Muju, Korea) and their authenticity was identified by Dr. Young Seung Joo at Department of Botany, Woosuk University (Jeonju, Korea), who is an expert in botanical identification. The rhizomes were dried at room temperature and then turned into powder. The powder was extracted using distilled water at 90°C for 12 h. Supernatants were collected following centrifugation at 10,000 ×g at 4°C for 20 min. Then the supernatants were lyophilized in a freeze-dryer. The yield was 22.1%. The contents of the total phenolic compounds in the extract were measured using Folin-Ciocalteu reagent and calculated using a standard (gallic acid) [12, 13], expressed in terms of mg gallic acid equivalents g^−1^. The total flavonoid content was also determined using the modified methods designed by Davis [14]. Rutin was used as a standard and the contents were expressed as mg rutin equivalents g^−1^.

Gastrodin was assigned as the indicator compound of GEB extracts. Its contents were measured using an Agilent 1100 series HPLC instrument (Agilent Technologies, Santa Clara, CA, USA) equipped with an autosampler (G1313A), column oven (G1316A), binary pump (G1312), DAD detector (G1315B), and degasser (G1379A). The contents of the indicated compounds in gastrodin were analyzed using a Luna 5u C18 100A (4.6 mm × 250 mm, Phenomenex, Torrance, CA, USA). The mobile phase consisted of distilled water, 0.1% formic acid (A), and acetonitrile (B) as solvents. The following gradients were used: 0–15 min, A : B 85 : 15 (v/v); 15–25 min, A : B 40 : 60 (v/v); 26 min, A : B 98 : 2 (v/v). The flow rate of the mobile phase, column temperature, injection volume, and wavelength for UV detection were 1.0 mL/min, 40°C, 10 *μ*L, and 270 nm, respectively.

### 2.2. Animal Care

Seven-week-old, male Sprague-Dawley rats (weighing 213 ± 9 g) were housed in individual cages in a controlled environment (23°C with a 12 h light/dark cycle). Animal care and surgical procedures adhered to the NIH Guide for the Care and Use of Laboratory Animals. This study was approved by the Animal Care and Use Committee of Hoseo University, Korea (2015-01). The rats were purchased from DBL (Yeumsung-Kun, Korea) and acclimated in our animal facility for 1 week.

### 2.3. Orchidectomy (ORX)

Following acclimation in our animal facility, rats were anesthetized by subcutaneous injection using a mixture of ketamine and xylazine (100 and 10 mg/kg body weight, resp.). In addition, gonadectomies were performed through a ventral incision in the scrotum [15]. Each testicle was exposed and removed after the ductus deferens was isolated and ligated. Incisions were subsequently closed and sutured. Sham-operated animals underwent the same procedures as above (i.e., acclimation, anesthetization, and surgery) but did not have their testes removed. Similar treatment in sham-operated animals was performed to measure possible stress induced by surgery. A single, subcutaneous injection of buprenorphine (0.03 mg/kg) was given postoperatively as an analgesic. Following the experiments, the presence of atrophy of the seminal vesicles was used as an indicator of proper testes excision. ORX rats were fed a diet of high fat for 1 week and then randomly divided into assigned groups.

### 2.4. Diet Preparation

Rats in all groups were fed high-fat diets which are known for exacerbating the symptoms of testosterone deficiency (compared to low-fat diets) [16–18]. The high-fat diet consisted of 37, 20, and 43 energy percent (En%) from carbohydrates, proteins, and fats, respectively. The high-fat diet was a semipurified, modified AIN-93 formulation for experimental animals [19]. The major carbohydrate, protein, and fat sources were starch plus sugar, casein (milk protein), and lard (CJ Co., Seoul, Korea), respectively. In the treatment groups, the lyophilized GEB extracts were used to supplement the high-fat diets (0.3% or 1%). Cellulose (0.3%) was used to supplement the diets of sham-operated and ORX-control rats. The GEB powder was mixed with a mixture of vitamins and minerals, as well as the sugar of the high-fat diet, until it was homogenous. Then this mixture was sifted to remove any lumps and combined with the appropriate amounts of starch, casein, and lard. It was sifted again and stored at 4°C. The nutrient compositions of the diets were the same. The amount of each supplement consumed (dosage) was calculated from the amount of food intake.

### 2.5. Experimental Design

After ORX, the rats were allowed to recover for 1 week with free access to water and a high-fat diet. At the beginning of week 2 post-ORX, 30 ORX rats were randomly assigned to the following groups: 0.3% GEB (GEB-L), 1% GEB (GEB-H), and 1% cellulose (ORX-control). Ten sham-operated rats were assigned to the normal-control group and fed a high-fat diet with 1% cellulose. Each group was fed its respective diet for 8 weeks. On every Tuesday at 10 a.m., following an overnight fast, the serum levels glucose, food intakes, and body weights were measured.

### 2.6. Measurement of Tail Skin Temperature

Tail skin temperatures were monitored during the sleep cycle every week of the experimental period using an infrared thermometer for small rodents (BIO-152-IRB, Bioseb, Chaville, France) [18]. Three measurements were made every 10 min and the average value was used as a single data point.

### 2.7. Measurement of Body Composition

The body composition of the rats was measured using a calibrated, dual-energy X-ray absorptiometer (DEXA; Norland pDEXA Sabre; Norland Medical Systems Inc., Fort Atkinson, WI, USA). The manufacturer supplied a phantom at week 7 of the experimental period. Animals were placed in a prone position and anesthetized with ketamine and xylazine (100 and 10 mg/kg body weight, resp.). Tape was used to keep their posterior legs maintained in a position of external rotation. The articulations of the hip, knee, and ankle joints were in 90° flexion. After the completion of scanning, the lean mass and bone mineral density of both legs and hips were measured using computer software connected to the DEXA instrument for small animals. The fat mass of the legs and abdominal areas were calculated using the same equipment.

### 2.8. Energy Expenditure Analysis by Indirect Calorimetry

On the second day following DEXA analysis, and after 6 h of fasting, energy expenditure was assessed at the beginning of the dark phase. The rats were acclimated for 30 min in a metabolic chamber (airflow = 800 mL/min) with a computer-controlled O_2_ and CO_2_ measurement system (BIOPAC Systems, Inc., Goleta, CA). After acclimation, their average oxygen consumption (VO_2_) and average carbon dioxide production (VCO_2_) were calculated over a period of 30 min. After these measurements, the data were averaged over 1 min intervals and the VO_2_ and VCO_2_ values were corrected for metabolic body size (kg)^0.75^. The respiratory quotient and resting energy expenditure were calculated using reported equations [20, 21]. Carbohydrate and fat oxidation were calculated from nonprotein oxygen consumption, as were their relative oxidative proportions. Carbohydrate and fat oxidation measurements were expressed as the amount of oxygen consumed per gram of substrate oxidized [16, 18].

### 2.9. OGTT and Collection of Samples

At the beginning of week 8 of experiments, an oral glucose tolerance test (OGTT) was performed on overnight-fasted animals by orally administering 2 g glucose/kg body weight. Blood samples were taken by tail bleeding at 0, 10, 20, 30, 40, 50, 60, 70, 80, 90, and 120 min after glucose loading. The serum levels of glucose were measured with a Glucose Analyzer II (Beckman, Palo Alto, CA). The serum levels of insulin were assessed at 0, 20, 40, 90, and 120 min using a radioimmunoassay kit (Linco Research, Billerica, MA). The average of the total areas under the curves (AUCs) for both glucose and insulin levels was calculated using the trapezoidal rule. At day 3 following OGTT, food was removed from the cages for 6 h prior to conducting an intraperitoneal insulin tolerance test (IPITT). Blood was collected every 15 min for 90 min after intraperitoneal injection of insulin (0.75 U/kg body weight). The glucose levels were measured from these blood samples.

Two days after the IPITT, the rats were anesthetized with ketamine and xylazine (100 and 10 mg/kg body weight, resp.) and blood was collected by abdominal cardiac puncture. Next, the serum was separated by centrifugation at 3,000 rpm for 20 min. After blood collection, human insulin (5 U/kg body weight) was immediately injected through the inferior vena cava to determine hepatic insulin signaling. Then the epididymal and retroperitoneal fat masses and uteri were removed and weighed. The uterus index was calculated as the uterus weight divided by body weight. Seminal vesicles were removed and their weights were also measured. Serum, liver, and skeletal muscle samples were collected and stored at −70°C for biochemical analysis.

Glucose and insulin levels were analyzed with a Glucose Analyzer II (Beckman Coulter, Palo Alto, CA, USA) and radioimmunoassay kits (Linco Research, Billerica, MA, USA), respectively. Insulin resistance was assessed with the homeostasis model assessment estimate of insulin resistance (HOMA-IR) using the following equation: HOMA-IR = fasting insulin (*μ*IU/mL) × fasting glucose (mM)/22.5. Testosterone levels were measured using ELISA kits (Enzo Life Sciences, NY, USA).

### 2.10. Blood Flow Measurement in Models of FeCl_3_-Induced Carotid Artery Thrombosis

At the end of the experiment and during operative procedures, body temperature was maintained at 37.0 ± 0.2°C using a heating pad. Rats were anesthetized with a mixture of ketamine and xylazine for surgery. One of the carotid arteries was exposed and a Doppler flow probe (TSD145, BIOPAC Systems, Inc., Goleta, CA) was placed on it. After stabilization for 3 min, vascular injuries were induced using a topical application of a 40% FeCl_3_-saturated filter paper for 4 min (2 × 2 mm). The filter paper was removed and the common carotid artery was washed with saline. A Doppler flow probe was placed on the artery exposed with FeCl_3_ and blood flow was measured continuously for 30 min with Laser Doppler Flowmetry (LDF100C-1, BIOPAC Systems, Inc., Goleta, CA). Arterial occlusion and complete occlusion were defined as decreased blood flow and cessation of blood flow in the carotid artery, respectively. Both the percentage peak platelet aggregation and time to remove platelet occlusion were measured.

### 2.11. Immunoblot Analysis

The liver was lysed with 20 mM Tris buffer (pH 7.4) containing 2 mM EGTA, 137 mM NaCl, 1% NP40, 10% glycerol, and 12 mM *α*-glycerol phosphate and protease inhibitors. Lysates containing equal amounts of protein (30–50 *μ*g) were used for immunoblotting with specific antibodies against protein kinase B (PKB/Akt), glycogen synthase (GSK)-3*β*, AMP kinase (AMPK), cAMP responding element binding protein (CREB), phosphoenolpyruvate carboxykinase (PEPCK), glucose transporter-2 (GLUT2), *β*-actin, phosphorylated forms of PKB^Ser473^, GSK-3*β*, CREB, and AMPK (Cell Signaling, Danvers, MA), as previously described [16]. The intensity of protein expression was measured using Imagequant TL software (Amersham Biosciences, Piscataway, NJ).

### 2.12. Statistical Analysis

Statistical analysis was performed using SAS software version 7 (SAS Institute, Cary, NC, USA). Results were presented as means ± standard deviation (SD) when the normal distribution was checked using Proc univariate. One-way analysis of variance (ANOVA) was used to separately assess the metabolic effects of the control, GEB-L, and GEB-H groups for the measurements that were taken once at the end of the experiment. The variables were measured over multiple time points and were analyzed with two-way repeated measures ANOVA. For this, time and group were independent variables and there was an interaction term between time and group. Significant differences in the main effects between the groups were identified by Tukey's test at *P* < 0.05.

## 3. Results

### 3.1. Contents of Total Polyphenols, Flavonoids, and Gastrodin

The GEB extract contained 410 ± 4 *μ*g/mg of total polyphenol, 257 ± 2 *μ*g/mg total flavonoids, and 3.68 ± 0.02 *μ*g/mg of gastrodin.

### 3.2. Serum Testosterone Levels and Tail Skin Temperature

At nine weeks after ORX, the ORX-control rats had significantly lower levels of testosterone, one-fifth of that of sham-operated rats (normal-control) (Table 1). GEB did not alter testosterone levels. Due to testosterone deficiency, the seminal vesicles in ORX-control rats were completely shrunk compared to normal-control. Specifically, the weights of seminal vesicles in the ORX-control rats were about 10-fold lower than in the normal-control (Table 1). GEB did not prevent the shrinkage of seminal vesicles (Table 1). The tail skin temperature was not significantly different among all the groups (Table 1). GEB did not change the tail skin temperature regardless of dosage (Table 1).

### 3.3. Body Composition and Energy Metabolism

ORX-controls gained less weight than normal-control during the 8-week experimental period. In addition, the relative ratio of epididymal fat pads to body weight was lower in ORX-control versus normal-control (*P* < 0.05). ORX rats that were given GEB-L gained more body weight versus ORX-control, but not as much as normal-control. However, body weight gain of the GEB-H group was not significantly different from that of ORX-control (Table 1). Epididymal fat mass was not significantly different among the ORX-control, GEB-L, and GEB-H although it was much higher in the normal-control group than ORX-control (Table 1).

**Figure 1:**
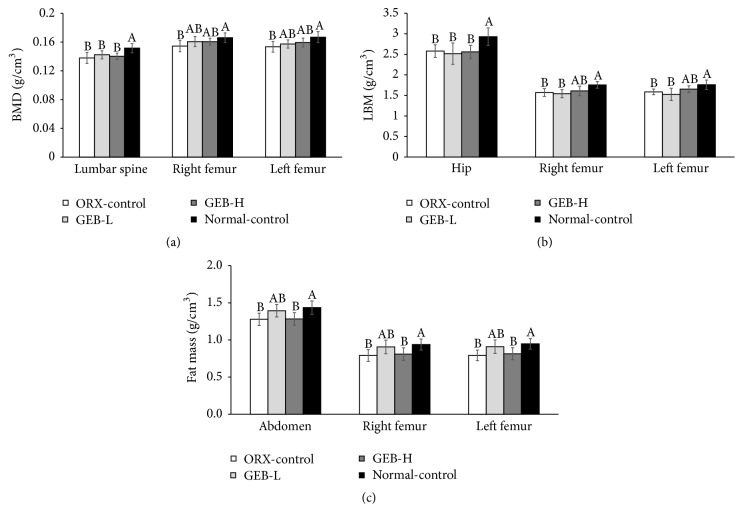
Bone mineral density (BMD), lean body mass (LMB), and fat mass (FM) at the end of experiment. Orchidectomized (ORX) rats consumed either 1% cellulose (ORX-control), 0.3% GEB (GEB-L), or 1% GEB (GEB-H) in a 43% fat diet for 8 weeks. Sham rats had 1% cellulose (normal-control). BMD (a) in the lumbar spine and femurs, LBM (b) of the hip and legs, and FM of the abdomen and legs (c) were measured by DEXA. Each bar and error bar represents the mean ± SD (*n* = 10). ^A, B, C^Values of the bars with different superscripts were significantly different among groups in Tukey's test at *P* < 0.05.

The BMDs of the lumbar spine and both femurs were much lower in the ORX-control than normal-control. The BMDs of the lumbar spine in the GEB groups were not significantly different from the ORX-control but those of both femurs in the GEB groups somewhat increased to the normal-control (Figure 1(a)). The LBM of the hips and legs also showed the same tendency as BMDs (Figure 1(b)): LBM in the hip and legs was lower in the ORX-control than the normal-control and GEB-H increased LBM in the legs. FM in the abdomen and leg was lower in ORX-control compared to normal-control. The FM of rats given GEB-H was similar to ORX-control (Figure 1(c)).

Daily energy intakes were not significantly affected by ORX or GEB (Table 1). Unlike energy intake, daily energy expenditure, as measured by indirect calorimetry, was much higher in ORX-control than in normal-control (Table 1). GEB-H, but not GEB-L, prevented the decrease in energy expenditure and was similar to that of normal-control (Table 1). RQs were not significantly different among the groups (Table 1). However, carbohydrate oxidation was lower in ORX-control than in normal controls, whereas fat oxidation exhibited the opposite pattern. GEB-L and GEB-H increased carbohydrate oxidation and decreased fat oxidation. The efficacy of GEB was higher in GEB-H versus GEB-L groups (Table 1).

### 3.4. Lipid Metabolism

ORX rats induced the disturbance of cholesterol metabolism (e.g., levels of HDL and LDL cholesterols and triglycerides) compared to normal-control. The levels of total cholesterol were not significantly different among the groups (Table 2). However, HDL cholesterol levels were lower in normal-control versus ORX-control; GEB prevented the decrease in levels of HDL in a dose-dependent manner in ORX rats. In contrast, the levels of LDL cholesterol were higher in ORX-control than normal-control; GEB-H lowered these levels (Table 2). The levels of triglycerides were lower in ORX-control than normal-control; GEB-L, but not GEB-H, increased these levels in ORX rats (Table 2).

### 3.5. Glucose Metabolism

Overnight fasting glucose concentrations were not significantly different among the groups (Table 2). However, the levels of fasting insulin were higher in normal-control versus ORX-control; GEB increased these levels in a dose-dependent manner (Table 2), but HOMA-IR, an index of insulin resistance, was not different among the groups (Table 2).

**Figure 2:**
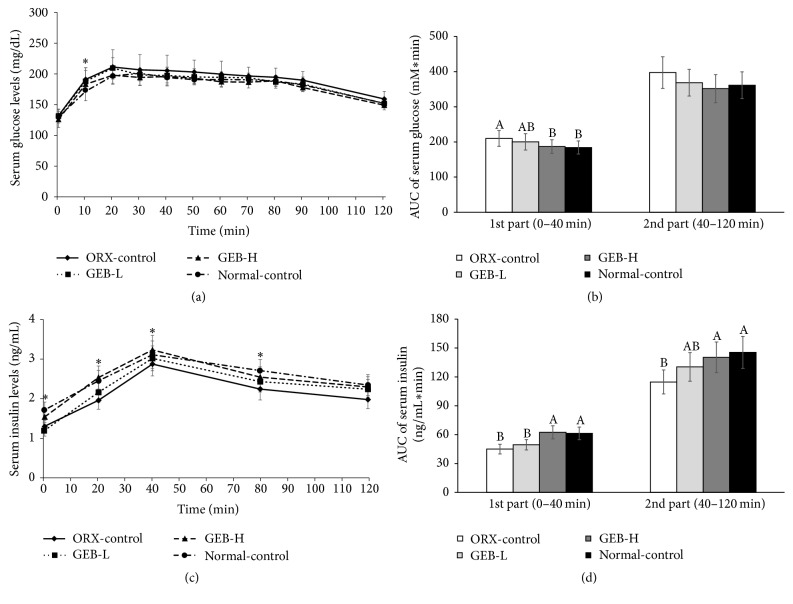
Serum glucose and insulin levels and area under the curve (AUC) of serum glucose and insulin during oral glucose tolerance test (OGTT). Orchidectomized (ORX) rats consumed either 1% cellulose (ORX-control), 0.3% GEB (GEB-L), or 1% GEB (GEB-H) in a 43% fat diet for 8 weeks. Sham rats had 1% cellulose (normal-control). Changes of serum glucose (a) and insulin (b) levels were measured after orally giving 2 g of glucose/kg body weight. The average of the area under the curve (AUC) of glucose (c) and insulin (d) during the first part (0–40 min) and second part (40–120 min) of OGTT. Each dot and bar and error bar represents the mean ± SD (*n* = 10). ^A, B, C^Values of the bars with different superscripts were significantly different among groups in Tukey's test at *P* < 0.05. ^*∗*^Significantly different among the groups at *P* < 0.05.

There was a little effect of ORX or GEB on glucose levels during the OGTT. At 20–30 min after oral glucose challenge serum glucose levels were higher in ORX-control than normal-control (*P* < 0.05). These levels slowly decreased with similar trends across all groups (Figure 2(a)). GEB-H had a similar peak glucose level compared to the normal-control group. The AUCs for glucose levels were initially (0–40 min) higher in ORX-control than normal-control. GEB-H was as low in ORX rats as it was in normal-control (Figure 2(b)). Later on (40–120 min), the AUC tended to be lower in normal-control, but it was not significantly different (Figure 2(b)). However, insulin levels were significantly lower in ORX-controls than in normal controls at every time point measured during OGTT. The levels were highest at 40 min in all groups (Figure 2(c)). GEB increased the levels of insulin during OGTT in a dose-dependent manner. The AUCs of insulin during the first (0–40 min) and second (40–120 min) phases were lower in ORX-control than normal-control (Figure 2(c)). The first-phase AUC of insulin levels increased in the order of ORX-control, GEB-L, GEB-H, and normal-control (*P* < 0.05). The second-phase AUC was higher in normal-control than ORX-control; GEB-H prevented the decrease in ORX rats (Figure 2(c)). This result suggests that ORX induced glucose intolerance due to lower insulin levels during OGTT.

**Figure 3:**
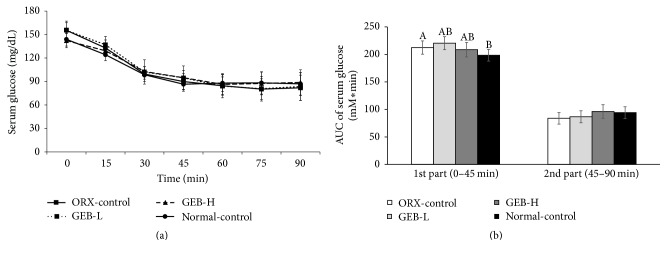
Changes of serum glucose concentrations during the intraperitoneal insulin tolerance test (IPITT). Orchidectomized (ORX) rats consumed either 1% cellulose (ORX-control), 0.3% GEB (GEB-L), or 1% GEB (GEB-H) in a 43% fat diet for 8 weeks. Sham rats had 1% cellulose (normal-control). ITT was conducted with intraperitoneal injection of 0.75 IU insulin/kg body weight and measured serum glucose concentrations in blood collected from the tail every 15 min for 90 min. Changes of serum glucose levels were measured during IPITT (a). The average of the area under the curve (AUC) of glucose (b) during the first part (0–45 min) and second part (45–120 min) of ITT. Each dot and bar and error bar represents the mean ± SD (*n* = 10). ^A, B, C^Values of the bars with different superscripts were significantly different among groups in Tukey's test at *P* < 0.05.

After an intraperitoneal injection of insulin, glucose levels were markedly lowered for 30 min. These levels further decreased and then increased from 60 to 75 min after injection (Figure 3(a)). At first, the AUC of glucose levels during IPITT was higher in ORX-control than normal-control. In contrast, it was not significantly different between the groups during the second phase. GEB did not change the AUC of either phase regardless of dosage (Figure 3(b)).

### 3.6. Hepatic Glycogen and Triglyceride Contents and Insulin Signaling

**Figure 4:**
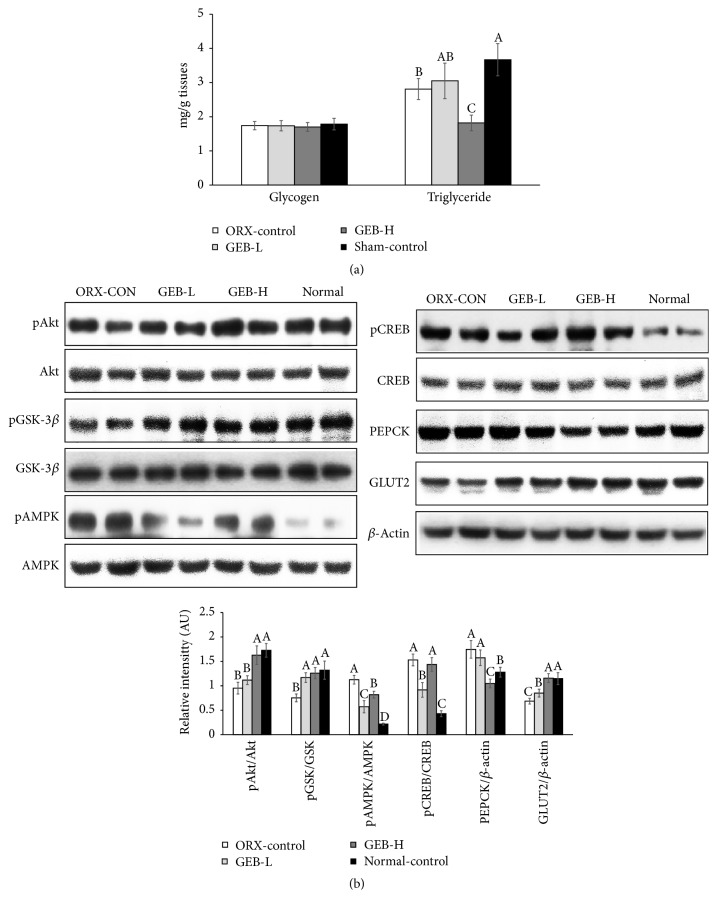
Hepatic glycogen and triglyceride levels and signaling pathways involved in glucose and lipid metabolism. Orchidectomized (ORX) rats consumed either 1% cellulose (ORX-control), 0.3% GEB (GEB-L), or 1% GEB (GEB-H) in a 43% fat diet for 8 weeks. Sham rats had 1% cellulose (normal-control). At the end of the experimental period, glycogen and triglyceride levels in the liver (a) and signaling pathways involved in glucose and lipid metabolism including hepatic insulin signaling (b) were measured. Each bar and error bar represents the mean ± SD (*n* = 10). ^A, B, C^Values of the bars with different superscripts were significantly different among groups in Tukey's test at *P* < 0.05.

The deposition of hepatic glycogen was not significantly different between ORX and normal-control; GEB did not alter the amount regardless of dosage (Figure 4(a)). However, hepatic levels of triglyceride were higher in normal-control than ORX-control; GEB-H decreased the deposition of triglyceride in the liver more to less than ORX-control (Figure 4(a)). Thus, GEB-H reduced lipid accumulation in the livers of ORX rats.

The phosphorylation of Akt and GSK, which are involved in hepatic insulin signaling, was attenuated in the ORX-control compared to normal-control; GEB-H prevented a decrease in their phosphorylation (Figure 4(b)). In contrast to insulin signaling, the phosphorylation of AMPK and CREB was lower in normal-control than ORX-control. GEB-L lowered the phosphorylation of AMPK and CREB, but not as much as in normal-control (*P* < 0.05). GEB-H did not decrease the phosphorylation of AMPK and CREB (Figure 4(b)). The expression of GLUT2, the major glucose transporter in the liver, was lower in ORX-control than normal-control; GEB prevented this decrease in ORX rats, indicating that glucose uptake might be lower in ORX-control versus normal-control (Figure 4(b)). In addition, PEPCK expression was not much different between the groups, but GEB-H lowered its expression (Figure 4(b)). Therefore, ORX rats had somewhat attenuated hepatic insulin signaling compared to normal-control. In addition, they had increased AMPK and CREB activities, which reduced triglyceride accumulation (*P* < 0.05). GEB-H improved hepatic insulin signaling in ORX rats but maintained the activities of both AMPK and CREB (*P* < 0.05).

### 3.7. Blood Flow by Removing Aggregated Platelets

**Figure 5:**
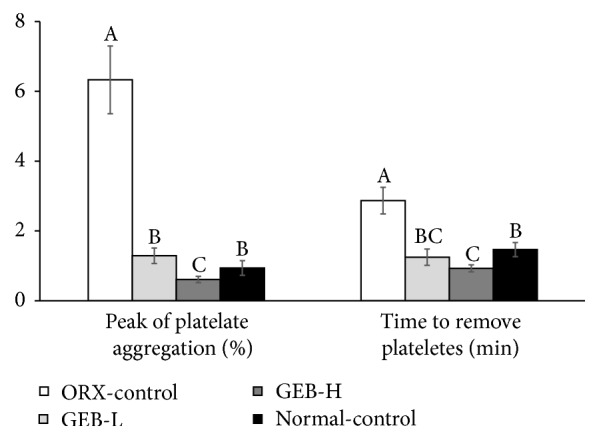
Blood flow measurement in FeCl_3_-induced carotid artery thrombosis models. Orchidectomized (ORX) rats consumed either 1% cellulose (ORX-control), 0.3% GEB (GEB-L), or 1% GEB (GEB-H) in a 43% fat diet for 8 weeks. Sham rats had 1% cellulose (normal-control). At the end of the experimental period, the blood flow was determined by the removal of vascular blockage. The percentage of peak platelet aggregation and time to remove platelet occlusion represented the degree of blow flow, and they were measured after a topical application of 40% FeCl_3_ to the carotid artery. Each bar and error bar represents the mean ± SD (*n* = 10). ^A, B, C^Values of the bars with different superscripts were significantly different among groups in Tukey's test at *P* < 0.05.

The arteries were occluded with platelets by FeCl_3_. Next, the peak percentage of occlusion and the time to remove the occlusion were measured to determine the degree of aggregation and blood flow, respectively (*P* < 0.05). Interestingly, the peak platelet aggregation was much higher in ORX-control than normal-control (Figure 5). It was reduced by GEB in a dose-dependent manner. In addition, the time to remove aggregations was much greater in ORX-control than normal-control. GEB prevented this increase in a dose-dependent manner (Figure 5).

## 4. Discussion

Aging and testosterone deficiency result in the development of cerebrovascular diseases such as stroke [5], but their effects may be mitigated by dietary and lifestyle changes. GEB is known to prevent brain-related diseases and improve energy and glucose metabolism. Previous studies have showed that after the consumption of GEB serum levels of gastrodin, a major component of GEB, are at the peak at 1 h [22]. Although we did not measure serum gastrodin levels, gastrodin might be absorbed into the circulation in the rats. In the present study, all ORX rats had significantly lower testosterone levels, one-fifth compared to the normal-control. Furthermore, seminal vesicles completely shrank in ORX-control. ORX altered body composition, energy, and lipid and glucose metabolism and blood flow was attenuated by ORX. Interestingly, ORX reduced triglycerides in the tissues and blood circulation, which could have benefits in cardiovascular diseases. However, it was detrimental to cholesterol and glucose metabolism, while also reducing blood flow. These results suggest that ORX increased the risk of metabolic diseases, including cardiovascular diseases and type 2 diabetes. GEB prevented the impairment of cholesterol and glucose metabolisms by reducing insulin levels in ORX rats. However, it did not prevent the decrease in triglycerides in tissues and circulation. Therefore, it could be a beneficial, functional food for reversing metabolic dysregulation in humans. This could also be accomplished without increasing serum testosterone levels in those with testosterone deficiency.

Interestingly, lean body mass and fat mass were both reduced in ORX-control. In particular, the reduction of body weight in ORX-control versus normal-control was associated with increased daily energy expenditure (particularly fat oxidation), while food intake remained unaltered. Several previous studies have demonstrated these same results, and that the loss of fat mass is reversed by testosterone treatment [23], and these results may not be surprising in growing animals deprived of an anabolic hormone. The fat loss in the liver and skeletal muscles was not associated with the expression of genes related to beta-oxidation. However, the increased thermogenic response was associated with elevated carnitine palmitoyl transferase-1 and uncoupling of protein-1 expression in brown adipose tissue [23]. It has been considered that brown adipose tissue is essentially nonexistent and without physiologic relevance in adult humans. However, recent studies have estimated that adults have over 50 g maximally stimulated brown adipose tissue that can account for up to 20% of daily energy expenditure [24, 25]. Therefore, the fat loss with increased fat oxidation might be related to an increased thermogenic response in testosterone-deficient humans. In the present study, GEB-H did not alter epididymal fat mass, while lean body mass was slightly increased in ORX rats. Fat oxidation was higher in rats given GEB-H versus normal-control but lower than in ORX-control. Carbohydrate oxidation was increased to similar levels in rats given GEB-H as well as normal-control. Thus, GEB-H maintained fat oxidation but increased carbohydrate oxidation in ORX rats. Previous studies [9, 26] have demonstrated that GEB (1 g/kg body weight) decreases fat accumulation and increases energy expenditure (particularly fat oxidation) in intact male rats. Thus, it might not further affect fat loss in ORX rats but might improve fuel usage from carbohydrates.

ORX changed lipid metabolism in the present study: ORX rats had lower levels of triglyceride and HDL cholesterol and higher LDL cholesterol in the circulation, compared to normal-control which was similar to the results of Movérare-Skrtic et al. [23]. In the present study, the reduction of triglycerides in the liver was related to increased phosphorylation of AMPK and CREB. The phosphorylation, and subsequent activation, of AMPK is associated with fat oxidation and also the elevation of CREB phosphorylation, which protects against hepatic steatosis. GEB-H also increased the phosphorylation of AMPK and CREB. Thus, ORX appears to modulate lipid metabolism although this remains controversial. GEB-H did not alter the reduction of triglyceride levels. Therefore, GEB exhibited antiobesity effects in ORX rats and improved cholesterol metabolism in the circulation.

The effects of ORX on glucose metabolism are controversial [22, 26, 27]. The present study also showed that ORX moderately suppressed glucose metabolism by decreasing insulin secretion. Overnight-fasted insulin levels were lower in ORX rats versus normal-control, while insulin resistance remained unchanged. GEB increased insulin levels in a dose-dependent manner. The level of glucose during the first phase (0–30 min) of glucose tolerance testing was significantly higher in ORX-control than in normal-control. During the second phase, these levels showed the same tendency, but it was not significantly different. Rats given GEB-H had lower levels of glucose during the first phase of OGTT compared to ORX-control. This decrease was similar to levels in normal rats. The levels of glucose were related to the levels of insulin. ORX lowered the levels of insulin in the first and second phases of OGTT, and GEB-H prevented a decrease in insulin levels. Previous studies have also showed that insulin levels in fasting states are lower in ORX rats than ORX rats treated with testosterone [23]. Cross-sectional studies have reported a close relationship between low levels of testosterone and type 2 diabetes, and low testosterone levels are a predictor of type 2 diabetes [27, 28]. Consistently, the present study showed that the AUC of glucose in the first phase of IPITT was also significantly higher in ORX rats versus normal-control. In addition, ORX-control had reduced glucose oxidation and lower insulin levels. Thus, ORX impaired glucose homeostasis by reducing glucose utilization. GEB improved glucose metabolism by increasing levels of insulin in both the fasting state and OGTT in ORX rats as much as in normal-control. Our results were consistent with a previous study that reported that GEB potentiates insulin secretion and cell mass in diabetic rats [29]. In addition, we found that GEB increased glucose oxidation in ORX rats as much as in normal-control. Thus, GEB-H may be a suitable therapeutic, functional food for improving glucose metabolism.

The FeCl_3_-induced thrombosis rat model is used to investigate changes in blood flow and the removal of platelet aggregation. This is an optimal technique because it is simple and reproducible. FeCl_3_ triggers an oxidative vascular endothelial matrix to induce platelet adhesion. Because blood flow is reduced when platelet aggregation is increased, blood flow was measured by the peak platelet aggregation and the time to remove platelets. Both were much higher in ORX-control than normal-control, while GEB reduced them in a dose-dependent manner. This indicates that ORX caused a reduction in blow flow. Mi et al. [30] reported that phenolic and furan type compounds in GEB have antiplatelet activities. Thus, GEB improved blood flow by preventing platelet aggregation.

## 5. Conclusions 

The findings of this study indicate that ORX reduced body fat and triglyceride levels in the liver, while increasing LDL cholesterol, lowering HDL cholesterol, reducing glucose metabolism, and lowering levels of insulin. Importantly, ORX also markedly reduced blood flow. These results suggest that ORX is associated with an increased risk for cardiovascular diseases although body fat was reduced. GEB had a positive impact on glucose and cholesterol metabolism and blood flow without affecting serum testosterone levels. Therefore, GEB may be a beneficial, functional food for decreasing the risk of cardiovascular diseases in humans with low testosterone levels. Further intervention studies need to support the effects of GEB on decreasing the risk of cardiovascular diseases in men with testosterone deficiency.

## Figures and Tables

**Table 1:** Serum testosterone levels and energy metabolism.

	ORX-control	GEB-L	GEB-H	Normal-control
Serum testosterone levels (pg/mL)	482 ± 38^B^	502 ± 46^B^	511 ± 41^B^	2,353 ± 218^A^
Seminal vesicle (mg)	39 ± 4^B^	42 ± 5^B^	41 ± 5^B^	416 ± 48^A^
Tail skin temperature (°C)	28.2 ± 0.15	27.9 ± 0.27	27.8 ± 0.30	27.6 ± 0.18
Final body weight (g)	413 ± 30^B^	436 ± 37^AB^	415 ± 33^B^	452 ± 33^A^
Body weight gain (g/8 weeks)	199 ± 17^C^	218 ± 18^B^	200 ± 13^C^	241 ± 15^A^
Epididymal fat mass (g)	7.42 ± 1.43^B^	8.15 ± 1.32^AB^	7.78 ± 1.25^B^	9.16 ± 1.39^A^
Food intake (g)	15.0 ± 1.4	15.3 ± 1.6	14.4 ± 1.4	14.6 ± 2.2
Energy expenditure (kcal/kg^0.75^/day)	115 ± 14^A^	109 ± 14^AB^	111 ± 12^A^	97.3 ± 12^B^
Respiratory quotient	0.79 ± 0.10	0.81 ± 0.10	0.82 ± 0.09	0.84 ± 0.10
Carbohydrate oxidation (mL/kg^0.75^/min)	3.4 ± 0.5^C^	4.0 ± 0.6^B^	4.4 ± 0.6^AB^	4.6 ± 0.6^A^
Fat oxidation (mL/kg^0.75^/min)	8.8 ± 1.0^A^	7.5 ± 0.9^B^	7.4 ± 0.9^B^	5.9 ± 0.8^C^

Orchidectomized (ORX) rats consumed either 1% cellulose (ORX-control), 0.3% GEB (GEB-L), or 1% GEB (GEB-H) in a 43% fat diet for 8 weeks. Sham rats had 1% cellulose (normal-control). Respiratory quotient = CO_2_ eliminated/O_2_ consumed. At the end of experiment, the parameters were measured and values are presented as mean ± SD. ^A, B, C^Values on the same row with different superscripts were significantly different among groups by Tukey's test at *P* < 0.05.

**Table 2:** Lipid and glucose metabolism.

	ORX-control	GEB-L	GEB-H	Normal-control
Total cholesterol (mg/dL)	99.9 ± 10.6	102.5 ± 11.3	99.6 ± 12.5	94.7 ± 10.7
HDL cholesterol (mg/dL)	38.5 ± 4.4^B^	40.8 ± 5.5^B^	47.5 ± 5.0^A^	46.1 ± 5.2^A^
LDL cholesterol (mg/dL)	53.7 ± 6.4^A^	52.7 ± 6.1^A^	43.9 ± 5.3^B^	38.3 ± 4.2^C^
Triglyceride (mg/dL)	38.6 ± 4.4^C^	45.2 ± 5.6^B^	41.2 ± 4.1^BC^	51.5 ± 6.2^A^
Glucose (mg/dL)	134 ± 12	131 ± 8	125 ± 14	125 ± 12
Insulin (ng/mL)	1.30 ± 0.17^B^	1.43 ± 0.23^AB^	1.57 ± 0.27^A^	1.54 ± 0.21^A^
HOMA-IR	6.3 ± 0.9	6.7 ± 1.0	7.0 ± 0.9	6.9 ± 1.0

Orchidectomized (ORX) rats consumed either 1% cellulose (ORX-control), 0.3% GEB (GEB-L), or 1% GEB (GEB-H) in a 43% fat diet for 8 weeks. Sham rats had 1% cellulose (normal-control). HOMA-IR, homeostasis model assessment estimate of insulin resistance. At the end of experiment, the parameters were measured and values are presented as mean ± SD. ^A, B, C^Values on the same row with different superscripts were significantly different among groups by Tukey's test at *P* < 0.05.

## Abbreviations

GEB: * Gastrodia elata* Blume
ORX: Orchidectomy
En%: Energy percent
GEB-L: The group fed a high-fat diet supplemented with 0.3% GEB
GEB-H: The group fed a high-fat diet supplemented with 1% GEB
ORX-control: The group fed a high-fat diet supplemented with 1% cellulose
VO_2_: Oxygen consumption
VCO_2_: Carbon dioxide production
RQ: Respiratory quotient
DEXA: Dual-energy X-ray absorptiometer
OGTT: Oral glucose tolerance test
AUCs: Total areas under the curves
IPITT: Intraperitoneal insulin tolerance test
HOMA-IR: Homeostasis model assessment for insulin resistance index
AMPK: AMP kinase
PKB/Akt: Protein kinase B
GSK: Glycogen synthase
PEPCK: Phosphoenolpyruvate carboxykinase
GLUT2: Glucose transporter-2
BMD: Bone mineral density
LBM: Lean body mass
FM: Fat mass
ANOVA: Analysis of variance.
